# Editorial: Suprastars of Chemistry

**DOI:** 10.3389/fchem.2022.932508

**Published:** 2022-06-06

**Authors:** Tangxin Xiao, Tony D. James, Victor Borovkov, Ronald K. Castellano, Chao Deng

**Affiliations:** ^1^ School of Petrochemical Engineering, Changzhou University, Changzhou, China; ^2^ Department of Chemistry, University of Bath, Bath, United Kingdom; ^3^ School of Chemistry and Chemical Engineering, Henan Normal University, Xinxiang, China; ^4^ Department of Chemistry and Biotechnology, School of Science, Tallinn University of Technology, Tallinn, Estonia; ^5^ Department of Chemistry, University of Florida, Gainesville, FL, United States; ^6^ College of Chemistry, Chemical Engineering, and Materials Science, Soochow University, Suzhou, China

**Keywords:** supramolecular chemistry, self-assembly, host-guest systems, metal coordination, macrocycle

The field of Supramolecular Chemistry has rapidly evolved over the last decades to the point where it now influences a diverse range of research areas, drives technological breakthroughs, and often results in breathtaking feats of ingenuity along the way. None of these advancements would be possible without the creativity and talent of supramolecular chemists throughout the world, and this Research Topic aims to celebrate those scientists as leading experts in the field—the “Suprastars.”

In this collection, excellent works cover fundamental research on self-assembly behavior, structure-property relationships, and chirality control, as well as attractive applications such as bioimaging, sensing, photodynamic therapy, and other related areas. The self-assemblies and supramolecular systems are facilitated by various kinds of dynamic non-covalent interactions, such as π-π interaction, (Hunter and Sanders, 1990; Shao et al., 2013; Fagnani et al., 2017; Xiao et al., 2019a; Mahl et al., 2022) macrocyclic host-guest interaction, (Yu et al., 2015; Xiao et al., 2019b; Roy et al., 2020; Xiao et al.; Fang et al., 2022) metal-coordination, (Sun et al., 2010; McConnell et al., 2015; Datta et al., 2018) and hydrophobic interactions (Chang et al., 2019; Xiao et al., 2019c) etc., which endow the corresponding materials with outstanding and highly desirable properties including reversibility, tunability, and stimuli-responsiveness.

In particular, macrocyclic host-guest chemistry is a hot topic in supramolecular chemistry on account of the continuous development of supramolecular macrocycles, such as crown ethers, (Pedersen, 1967; Price and Gibson, 2018; Xiao et al., 2021) cyclodextrins, (Szejtli, 1998; Harada et al., 2014) cucurbiturils, (Lagona et al., 2005; Masson et al., 2012; Ni et al., 2014; Barrow et al., 2015; Murray et al., 2017; Chen et al., 2022; Huang et al., 2022) calixarenes, (Böhmer, 1995; Guo and Liu, 2014), and pillararenes (Xue et al., 2012; Strutt et al., 2014; Ogoshi et al., 2018; Xiao et al., 2018; Xiao et al., 2019d; Wan et al., 2022). Fullerenes remain important compounds with promising applications in biomedical research but their hydrophobicity limits deployment in the body. Zhang et al. developed a water-soluble supramolecular nanoformulation based on a deep cavitand calixarene system (SAC4A), which is able to host fullerene *via* a simple grinding approach. The system enables efficient activation of reactive oxygen species and can be used as a potential photodynamic agent. Duan et al. developed a pillararene-indicator displacement system. The water-soluble pillar [6]arene macrocycle can greatly enhance the fluorescence of safranine T (ST) due to host-guest induced twisted intramolecular charge-transfer. The system can be used as a turn-off sensor for caffeine in water due to guest exchange.

As another important property, manipulating molecular chirality has been attracting significant attention. Liu et al. synthesized several new chiral pillar [4]arene [1]quinone derivatives, which showed unique chiroptical properties. Notably, the benzene sidearm attached pillar [4]arene [1]quinone derivative exhibited solvent- and complexation-driven chirality switching. Hemicucurbiturils are chiral macrocyclic hosts similar to the cucurbituril macrocycles and their monomeric units are connected by one row of methylene bridges. Ustrnul et al. systematically studied the complexation between cyclohexanohemicucurbit [n]urils and eighteen polar organic guests.

Metal-coordination is another important non-covalent interaction that is usually employed by supramolecular chemists to construct discrete entities including metallacycles (Chakrabarty et al., 2011) and metallacages, (Mal et al., 2009; Sun et al., 2010) as well as infinite architectures such as supramolecular polymers (Winter et al., 2016) (Winter and Schubert, 2016) and metal-organic frameworks (MOF) (Stock et al., 2012), (Stock and Biswas, 2012) etc. One particularly interesting application of supramolecular analytical methods is in the assessment of food freshness. As such, Lyu et al. developed a self-assembled colorimetric chemosensor array for the qualitative and quantitative detection of sulfur-containing amino acids. It is noteworthy that the sensor is based on the reversible coordination between off-the-shelf catechol dyes and Zn^2+^, which offered obvious color changes in the presence of the analytes.

Helicates are another class of interesting metallo-supramolecular architecture that may have potential biological applications. Lisboa et al. and Lisboa et al. synthesized two new di (2,2′-bipyridine) ligands, which can self-assemble into specific metallo-supramolecular [Fe_2_(**L**)_3_](BF_4_)_4_ cylinders. Moreover, *in vitro* cytotoxicity assays showed that these helicates were active against several cancer cell lines.

Metal-organic cages (MOCs) also belong to metallo-supramolecular architectures and their cavities are capable of binding guest molecules. In a mini review, Diaz and Lewis focused on the structural flexibility in MOCs and summarized typical examples of MOCs reported in recent years.

Supramolecular polymers based on coordination interactions are polymeric arrays in which the building blocks are brought together *via* metal coordination. Mackenzie et al. synthesized a 1D coordination polymer consisting of silver(I) ions bound to a [2.2] paracyclophane scaffold. The coordination polymer was fully characterized by single crystal X-ray analysis and shows strong blue fluorescence.

MOFs provide an effective template for polymerization of polymers with precisely controlled structures within the nanochannels. Wonanke et al. explored the interaction of styrene and 3,4-ethylenedioxythiophene (EDOT) at the surface and in the nanopore of a Zn-MOF. They discovered that the monomer-MOF interaction is strongest inside the nanochannels and increases with the number of monomers.

Given the π-π interaction between large polycyclic aromatic hydrocarbon (PAH) molecules and fullerene, Gover et al. developed a new combined TEM and MALDI-TOF mass spectroscopic approach to detect different nano-architectures.

Hydrophobic interactions predictably drive amphiphiles to form nano-assemblies in water. Ranathunge et al. linked a hydrophobic dye TRPZ to a hydrophilic dendron *via* azide-alkyne Huisgen cycloaddition to prepare an amphiphilic system TRPZ-bisMPA, which can further form nanoparticles in aqueous media. The authors reported that TRPZ-bisMPA nanoparticles are of low cytotoxicity, hence being suitable for bioimaging.

In another work, Magna et al. developed a facile and rapid method to achieve chiral porphyrin films on a glass support. They systematically studied the solvent effect and glass substrate on the film formation.

In summary, this Research Topic has highlighted the most advanced and cutting-edge developments in supramolecular chemistry led by “Suprastars” from all over the world. By taking the advantage of dynamic supramolecular interactions, a series of functional self-assembled nano-architectures have successfully been constructed and been fully evaluated. More interestingly, the intersection of supramolecular chemistry and other disciplines has yielded new characterization methods and novel functional materials. We believe that more and more “Suprastars” in the field of supramolecular chemistry will emerge and as a result more and more impact rich research continue to evolve.
